# Wildfire brigade members and wildland firefighters on the frontline of climate change: An essential, strategic, and vulnerable role workforce in the era of intensifying wildfires

**DOI:** 10.1097/EE9.0000000000000465

**Published:** 2026-02-09

**Authors:** Elizeu Chiodi Pereira, Alda Neis Miranda de Araújo, Kamila de Almeida Piai, Vinicius Guimarães Ferreira, Fernando Viana Rodovalho, Sandra Cortés, Dwan Vilcins, João Paulo Teixeira, Isarita Martins, Sandra Hacon, Rafael Junqueira Buralli, Kelly Polido Kaneshiro Olympio

**Affiliations:** a Departamento de Saúde Ambiental, Faculdade de Saúde Pública, Universidade de São Paulo, São Paulo, Brasil; b Expossoma e Saúde do Trabalhador - eXsat (Exposome and Worker’s Health), Universidade de São Paulo, Faculdade de Saúde Pública, São Paulo, Brasil; c Deutsche Gesellschaft fuer Internationale Zusammenarbeit (GIZ), Brasília, Brasil; d Escuela de Salud Publica, Pontificia Universidad Catolica de Chile, Santiago, Chile; e Child Health Research Centre, The University of Queensland, South Brisbane, Australia; f National Institute of Health Dr. Ricardo Jorge, Porto, Portugal; g Faculty of Pharmaceutical Sciences, Federal University of Alfenas, Minas Gerais, Brasil; h Escola Nacional de Saúde Pública Sergio Arouca, Fundação Oswaldo Cruz, Rio de Janeiro, Brasil; i Departamento de Política, Gestão e Saúde, Faculdade de Saúde Pública, Universidade de São Paulo, São Paulo, Brasil

## Understanding the challenge

It is unacceptable that individuals who risk their lives protecting ecosystems, communities, and even the most remote of territories are often exposed to high-risk jobs and overlooked by legislation and public policies. The more wildfire workers operate on the frontline of climate change, the greater are the risks they face.

In Brazil, wildfire brigade members (WBM) and wildland firefighters (WF) play a key role in wildfire control and prevention. WBM include temporary workers hired by federal environmental agencies (typically for 6–24 months), volunteers, and traditional community members (riverine, quilombolas, Indigenous, and women of reproductive age). Despite the job’s complexity, many come from socially vulnerable groups, facing precarious work conditions, limited rights, poor access to health care, and weakened health.

Volunteers and community brigade members routinely engage in this work, frequently lacking social and labor protections, and adequate access to essential personal protective equipment (PPE), including masks and other safety devices.^1^ Female workers face even greater health challenges, including the fact that PPE often do not fit well in women, contributing to an unsafe work.^2^ Moreover, urinary tract infections and inadequate menstrual management lead to workforce absenteeism.^3^ The lack of state support and occupational health measures heightens physical and mental health risks. Yet their occupation remains unrecognized as a profession in Brazil, where bills establishing formal employment, career paths, minimum wage, and hazard pay remain stalled in Congress. In 2025, approximately 4300 temporary hires under the Ministry of Environment and Climate Change were expected to work alongside about 5000 volunteer and community brigade members.^4,5^

By contrast, WF are affiliated with military institutions and hold formal contracts, with greater job stability and a career path within these organizations. Both groups routinely face exposure to a wide range of physical, chemical, biological, and ergonomic hazards. These risks are compounded when working in remote areas, involving activities that demand substantial physical exertion in carrying equipment and suppressing wildfires.^1^

As an example, Bomberos de Chile is a nationwide volunteer organization comprising local and intercommunal fire departments. It responds to natural and human-made emergencies, including fires, accidents, rescues, and hazardous material spills, entirely on a volunteer basis. Funding comes mainly from the government, supplemented by donations and paid nonemergency services.^6^ Chile has over 50,000 active volunteer firefighters, including operational and support members. All volunteers are covered by a special health program that provides medical and psychological care for work-related injuries or illnesses, aligned with the National Health Strategy, which pursues universal coverage, equity, and protection for vulnerable groups, including emergency responders.^7^

Before the misconception arises that wildfires are a localized issue, it is important to consider the global perspective, where millions of people are affected annually due to the increased presence of air pollutants.^8,9^ For example, the United States lost almost 3 million acres in 2023.^10^ A similar scenario can be seen in Australia, where, between 2016 and 2021, almost 47 million hectares of forests burned.^11^ In Portugal, during the 2020–2023 period, 24 megafires were recorded, and an area of over 600,000 hectares was destroyed by fire events.^12^ In Greece, almost 200,000 hectares burned in 2023.^13^ Tropical regions of South America have been experiencing intense wildfire activity in the second half of February, following increased wildfire activity in Chile and Argentina.

These episodes are mostly driven by human activities, which cause widespread environmental damage and reduce socio-biodiversity, with vulnerable communities experiencing disproportionately higher losses and adverse consequences, despite contributing less to these impacts.^14^ WBM and WF are pivotal in preventing and containing wildfires, reducing the harmful health effects in exposed populations. Against this backdrop, the question remains: why is the protection of the acute and chronic health of these workers still neglected?

Smoke from wildfires contributes to an increased risk of developing cardiorespiratory diseases and cancers, impacting around 70 million people worldwide and causing considerable socioeconomic damage to regions affected.^15–19^ Moreover, roughly 1.53 million people die as a result of pollution caused by wildfires worldwide, with cardiovascular diseases representing the most prevalent related cause of death.^20^ Sub-Saharan Africa accounts for 40% of these deaths,^20^ with the majority of Disability-Adjusted Life Years linked to the almost 7.5 million wildfire events worldwide, especially on the Asian continent.^21^ In the 2023 report of the Lancet Count Down for Latin American countries, the number of days in which people were exposed to very high and extremely high fire danger in the 2013–2022 period increased, compared with 2001–2010, with an average of 1 additional day of exposure per person. The highest increases were seen in Chile, Venezuela, Argentina, Colombia, and Brazil.^22^

This is the haphazard scenario in which WBM are situated. For many individuals, this represents their livelihood and the way they support their families. This group works under conditions that clearly violate the minimum standards for decent work of the International Labour Organization, incorporated into the United Nations Sustainable Development Goals (SDG 8),^23,24^ and is exposed to a wide range of environmental pollutants and health risks as a result of their role in protecting forests and the communities. However, all workers must have access to workplace safety, fair pay, social protection, defined work periods, adequate training, and gender equity.^25^

Despite recent increase in intensity and occurrence of wildfires globally due to climate change, data on occupational safety and health of WBM and WF are still scarce and much needed. Limited data on WF derived from countries such as Portugal, the United States, Australia, and Canada, while low- and middle-income countries, with potentially higher exposures, remain understudied.^26,27^

This commentary demonstrates, through ongoing research in the Brazilian Amazon, that protecting the health of wildfire brigade members and wildland firefighters protects the health and well-being of everyone. It outlines the working conditions of these workers and the occupational risks to which they are exposed, reporting preliminary findings.

## Occupational risks for wildfire brigade members and wildland firefighters

Professionals involved in wildfire suppression face highly precarious working conditions, leaving them vulnerable to a range of health risks. Despite their exhausting and essential work, they lack insurance, paid leave, social security, and other labor rights. Globally, PPE for WF and brigade members is often inadequate and insufficient. Respiratory protection may not be fit-tested or suitable for wildland conditions, clothing and boots can be too heavy for remote work, and access to protective gear is frequently limited by cost or logistics.^28^

In many territories, these workers also assume the role of community leaders and fire managers. This responsibility entails planning, organizing, and coordinating prevention and mitigation strategies by mobilizing knowledge, people, and resources to protect lives and landscapes. The role is complex and highly strategic, but is often performed with no institutional recognition. This point is especially poignant with respect to indigenous brigades that engage in traditional fire management. As previously observed in Australia, the interruption of Aboriginal practices resulted in a collapse of plant species and resource availability, highlighting the strategic, organized, and coordinated planning carried out by these individuals.^29^

In Brazil, wildfires are becoming more frequent. By 2023, about 200 volunteer and community brigades had been identified for wildfire prevention and control, approximately 23% of which were male and female Indigenous workers. Intensifying windstorms, rising temperatures, heat waves, and prolonged droughts now alter wildfire frequency and behavior, often making prevention and mitigation efforts unfeasible, especially within Indigenous lands.

In line with this discussion, a recent report by the Brazilian Ministry of Health has underscored the multiple occupational risks faced by these WBM. Beyond the immediate danger of accidents, these workers are exposed to physical hazards, chemical hazards, biological hazards, and ergonomic and psychosocial stressors.^30^

Evidence confirms harmful health effects from occupational exposure among these workers. Key findings include genotoxic and oxidative stress, DNA damage, increased cell death, elevated toxic elements in biological samples postshift, and up to 23-fold higher polycyclic aromatic hydrocarbon levels than those found in the general population, along with increased defense cell counts.^31–35^

The work of structural firefighters is often associated with the development of several types of cancers, and, for this reason, the occupation was classified as carcinogenic in 2023 by the International Agency for Research on Cancer.^36^ This classification may also extend to WBM and WF, who are exposed almost daily to some of the same classes of chemical compounds as firefighters. However, it is important to emphasize that unlike what is observed in other countries, Brazilian WBM do not use foams for wildfire suppression, which are known to often contain high concentrations of per- and polyfluoroalkyl substances.^37^

This situation underscores the pressing need to monitor these workers more closely and to conduct studies evaluating exposure to contaminants present in wildfire smoke to obtain a more accurate estimate of the associated risk and thus better promote and protect the health of these individuals.

## Research approach to wildfires in the Brazilian Amazon

To address the dearth of information on the health of WBM, between July and December 2024, we collected biological samples (blood, urine, saliva, and buccal swabs) from WBM and a control group to evaluate biomarkers of exposure, genotoxic and oxidative stress, lipid profile, metabolic and inflammatory markers, stress-related hormones, and untargeted metabolomic and proteomic profiles. Environmental characterization, including inhaled air and fire ashes, and an ergonomic work analysis on a subsample of workers are also conducted (Ethics:74890123.0.0000.5421).

The study is taking place in Pará, Brazil’s second-largest state, covering approximately 1.2 million km^2^ with 9 million inhabitants.^38^ The host of COP30, Pará is part of the Amazon’s “Deforestation Arc,”^39^ and accounted for 42% of Amazon deforestation between 2009 and 2019.^40^ The state is driven by agribusiness expansion, illegal deforestation and gold mining, land grabs, intensive use of pesticides and other chemicals, affecting vulnerable populations. We have included 411 participants with biological samples collected from 370 individuals (131 wildfire brigade members and 239 controls).^41^

Preliminary assessment of ongoing research suggests that WBM experience dehydration, systemic inflammation, and metabolic disturbances, particularly in relation to wildfire exposure intensity.^42,43^

## Recommendations and final remarks

WMB and WF are frontline workers increasingly needed amid the climate crisis and rising wildfire frequency. Protecting them is vital for effective climate mitigation and fire management, safeguarding public health and well-being. Although epidemiological data are scarce, these vulnerable workers face numerous occupational risks with potential acute and chronic effects. Addressing risk disparities is essential, particularly for growing brigades formed by traditional communities and women of reproductive age, often volunteering. Women require specific health monitoring, especially regarding heat exposure and physical strain. All brigade members must be protected from hazardous exposures and guaranteed their right to decent work, regardless of employment status.

Protecting these workers demands coordinated action from governments, employers, academia, and NGOs. These efforts include capacity building, integrated incident management, and professional recognition to regulate the profession. Policies must reduce structural inequalities by ensuring basic rights, comprehensive health care, occupational safety, surveillance, and recognition of wildfire brigades’ essential role in climate mitigation and environmental protection. Agencies must provide and develop appropriate PPE for extreme conditions that protect these professionals from both fine particulate matter and gases, as well as ensure rest, food, water, and clean air during major events. Integrating health protection with equal rights and recognition is vital to safeguard and value these workers and their communities.

Finally, studies providing a foundational understanding of the occupational risks of these workers are essential. Future research should focus on longitudinal follow-up for both WF and WBM. Collaborations with international groups, experienced in biomonitoring and fire exposure, are encouraged to help promote comparative studies, standardize protocols, and enhance researcher training. Studies should be co-designed and contain a direct translational element to ensure that WF and WBM conditions are improved from their involvement in research studies. Such partnerships can support global efforts toward protecting the health and labor rights of these workers, while ensuring decent work and empowerment of the most vulnerable amid the growing challenges of climate change.

## Conflicts of interest statement

The authors declare that they have no conflicts of interest with regard to the content of this report.

## ACKNOWLEDGMENTS

*We thank all researchers involved in writing this commentary. We also extend our gratitude to the agencies linked to the Brazilian Ministry of Environment and Climate Change (Instituto Chico Mendes de Conservação da Biodiversidade and Instituto Brasileiro do Meio Ambiente e dos Recursos Naturais Renováveis) for their support during the biological sample collections of the wildfire brigade members. We extend our acknowledgments to the Institute for Ecological Research (IPÊ), especially to Dr. Ângela Pellin for her support in coordination and collaboration with the field team, which enabled the identification of volunteer brigades in the state of Pará. Finally, we appreciate the participation of those in the Brazilian Amazon, including frontline fire combat workers and the general population who made up the environmental control group.*
